# Urban school neighbourhoods dominated by unhealthy food retailers and advertisements in Greater Tunis: a geospatial study in the midst of the nutrition transition

**DOI:** 10.1017/S1368980023002860

**Published:** 2024-01-03

**Authors:** Christelle Akl, Nehmat El-Helou, Gloria Safadi, Aline Semaan, Aya El Sammak, Tarek Trabelsi, Sonia Sassi, Chaza Akik, Jalila El Ati, Pierre Traissac, Hala Ghattas, The SCALE Research Group

**Affiliations:** 1 Center for Research on Population and Health, Faculty of Health Sciences, American University of Beirut, Beirut, Lebanon; 2 Department of Public Health, Institute of Tropical Medicine, Antwerp, Belgium; 3 INNTA (National Institute of Nutrition and Food Technology), SURVEN (Nutrition Surveillance and Epidemiology in Tunisia) Research Laboratory, Tunis 1007, Tunisia; 4 MoISA – University of Montpellier, CIRAD, CIHEAM-IAMM, INRAE, Institut Agro, IRD, Montpellier, France; 5 Department of Health Promotion, Education, and Behavior, Arnold School of Public Health, University of South Carolina, Columbia, SC 29208, USA

**Keywords:** School neighbourhoods, Retail food environments, Food advertisements, Geographic information system, Low- and middle-income

## Abstract

**Objective:**

Food environments are a major determinant of children’s nutritional status. Scarce evidence on food environments exists in low- and middle-income countries (LMIC). This study aims to fill this gap by documenting the obesogenicity of food environments around schools in Greater Tunis, Tunisia – an LMIC of the Middle East and North Africa region with an ongoing nutrition transition and increasing rates of childhood obesity.

**Design:**

In this cross-sectional study, we assessed built food environments around fifty primary schools. Ground-truthing was performed to collect geographic coordinates and pictures of food retailers and food advertisement sets within an 800-m road network buffer of each school. Retailers and advertisement sets were categorised as healthy or unhealthy according to a NOVA-based classification. Associations between school characteristics and retailers or advertisement sets were explored using multinomial regression models.

**Setting:**

Greater Tunis, Tunisia.

**Participants:**

Random sample of fifty (thirty-five public and fifteen private) primary schools.

**Results:**

Overall, 3621 food retailers and 2098 advertisement sets were mapped. About two-thirds of retailers and advertisement sets were labelled as unhealthy. Most retailers were traditional corner stores (22 %) and only 6 % were fruit and vegetable markets. The prevailing food group promoted was carbonated and sugar-sweetened beverages (22 %). The proportion of unhealthy retailers was significantly higher in the richest *v*. poorest areas.

**Conclusions:**

School neighbourhood food environments included predominantly unhealthy retailers and advertisements. Mapping of LMIC food environments is crucial to document the impact of the nutrition transition on children’s nutritional status. This will inform policies and interventions to curb the emergent childhood obesity epidemic.

Over the last decades, childhood overweight and obesity have dramatically increased in low- and middle-income countries (LMIC), with the Middle East and North Africa (MENA) region experiencing one of the largest increases in childhood obesity rates, reaching around 20 % in 2016^(1)^. Complex and intertwined factors that span the socio-ecological model have been shown to influence children’s diet and nutritional status^(2)^. Among these factors, the intermediate structures or meso-level factors, such as school neighbourhood food environments, play a major role in shaping children’s food choices and subsequently weight status^(3)^.

Food environment is defined as ‘the interface within which people interact with the wider food system to acquire and consume foods’ ^((4), p, 95)^. As it includes the multitude of food options available to people in their environments, it can influence food choices, purchasing behaviours and dietary intake – all of which have implications on the development of obesity and other diet-related non-communicable diseases at all stages of life^(4,5)^. Promoting healthy food environments is a public health priority – it is among the objectives of the United Nations Decade of Action on Nutrition 2016–2025 in fighting malnutrition^(6)^ and has implications on a wide range of nutrition-related Sustainable Development Goals (SDG) including SDG 2 ‘Zero hunger’ and SDG 3 ‘Good health and wellbeing’^(7)^.

Food environments might influence food habits through direct access to foods or through food cues and desire^(8,9)^ – this influence is even more marked among schoolchildren. Schools and their neighbourhoods are key sites that influence food choices as children spend a large portion of their day in school and are more autonomous in their food choices^(10,11)^. Beyond food provided within the school itself, children might buy snacks from outlets in the vicinity of schools during recess or on their way to and from school^(12–14)^. One study conducted in Scotland showed that about 14 % and 30 % of children from primary and secondary schools, respectively, purchased food from outlets on their way to/from school^(14)^. Food advertisements can also influence children’s food choices within school hours and after^(15)^. Some evidence shows that fast-food restaurants and food advertisements tend to cluster around schools^(16,17)^ with sugar-sweetened beverages and high-fat foods being among the most advertised products^(17,18)^. It has been hypothesised that school neighbourhood food environments can facilitate exposure and access to low-cost, energy-dense and ultra-processed foods – all of which might encourage children to choose, purchase and consume unhealthy food. Conversely, food environments that mainly offer and promote healthy and nutritious food choices (such as fruits, vegetables or unprocessed/minimally processed foods) might improve children’s diet quality and weight status^(19,20)^.

Socio-economic patterning of built food environments has also been documented in several high-income countries (HIC). For instance, density of fast-food restaurants was higher around schools located in disadvantaged areas as compared with those in more advantaged ones^(21,22)^. Similarly, advertisements promoting unhealthy foods were more frequent in areas with high levels of socio-economic deprivation as compared with those with lower levels^(23,24)^. Other studies conducted in HIC found minor or no significant associations between area-level socio-economic status and type of food retailers or advertisements^(25,26)^.

The available literature on built food environments highlights the multitude of metrics (e.g. count, count per area, proximity, etc.), geographic boundaries (e.g. areal, person-centric or buffer measures) and classification systems used in food environments research^(27,28)^. Also, most food retailer constructs used in the literature (e.g. convenience stores, fast-food restaurants, grocery stores) are designed for HIC and are often inappropriate for LMIC, where many traditional food retailers do not fit within these pre-defined constructs^(28)^. This hinders comparability across studies and might explain the inconsistent results observed in research looking at associations between food environments and children’s nutritional status^(5)^.

While there are considerable studies from HIC on school neighbourhood food environments, less evidence exists in LMIC – with most studies being of low quality^(29)^. To the best of our knowledge, few studies have assessed neighbourhood food environments in the MENA region^(30,31)^ and none have assessed these using geospatial methods^(29)^. This is a considerable research gap given that the last decades have been marked by substantial changes in food systems and dietary behaviours in the MENA region with multiple countries experiencing rapid nutrition and epidemiological transitions^(32)^. Tunisia is a lower-middle-income country of the MENA region having experienced rapid rates of economic development and urbanisation. Childhood overweight rates in Tunisia have doubled over the past decades^(1)^ with estimates reaching 29 % in boys and 32 % in girls among 6- to 9-year-old children living in Greater Tunis^(33)^.

This study aims to fill this important research gap by providing a comprehensive assessment of the quality of the built (i.e. external) food environment around Tunisian primary schools. The study objectives are to (1) map all types of food retailers and food advertisements present around primary schools in urban areas of Tunisia; (2) classify these food exposures as healthy, unhealthy or mixed, using a typology derived from the NOVA classification^(34)^; (3) describe food retailers and advertisements using count, density and proximity measures and (4) investigate whether these food exposures differ by school (geographic and/or socio-economic) characteristics.

## Methods

### Study site and sample

This study is part of a larger project entitled ‘School and community drivers of child diets in Arab cities; identifying levers for intervention (SCALE)’, which aimed to investigate school and community-level drivers of children’s food choices in two Arab cities: Greater Tunis in Tunisia and Greater Beirut in Lebanon^(35)^. In the present study, we focus on the Tunisian part of the project. Tunisia has 11 million inhabitants with two-thirds of the population living in urban areas. The study area is the ‘Greater Tunis’ region, which includes the four ‘Governorates’ of Ariana, Ben Arous, Manouba and Tunis (the capital city)^(36)^. A cross-sectional survey used a random sample of fifty primary schools proportionally stratified by type of school (public (70 %) *v*. private (30 %)); fifty children were then randomly selected within each school. The sample size of fifty schools was based on the sample size calculation conducted for the SCALE project – further details can be found elsewhere^(35)^.

### School neighbourhood food environment

#### School neighbourhood unit and mapping protocol

All food retailers and food advertisements present within an 800-m road network of each school were mapped through ground-truthing, that is, in-person mapping with direct observation and measurement/assessment on the ground of food exposures^(37)^. Ground-truthing was performed as (1) no commercial or governmental lists on food retailers are publicly available in Tunisia and (2) field observation is considered the gold standard to document all existing food exposures in neighbourhoods^(4)^. We opted for an 800-m road network buffer around schools as it corresponds to the distance that an average school-age child can walk within 10 min^(38)^. To draw the buffers, a governmental open-source map of Greater Tunis main roads (*n* 812) was used as the base map. Manual drawing of street-level roads was conducted using both Google Earth and street map view base maps on ArcGIS (ArcGIS 10, ESRI Inc.); 7357 streets were thus added to the map.

Data collectors were given mobile phones with integrated geographic positioning system and asked to collect geographic coordinates and pictures of all food retailers (including informal ones) and food advertisements present within the 800-m road network buffers of each of the fifty schools using two applications: Collector Classic**®** and Survey123**®** (ESRI Inc.). Pictures of food retailers and advertisements were taken as a verification step for quality assurance. The geographic coordinates of schools were also collected. The neighbourhood mapping was conducted from September till October 2020, which coincides with the re-opening of schools after the COVID-19 lockdown was lifted in Tunisia. Mapping was also conducted during normal school hours to capture regular food environments on school days. Training of data collectors, piloting of data collection tools, field monitoring and verification of data entered after each field visit were all conducted to collect high-quality data. Reporting of this study method is based on the GeoFERN framework^(27)^.

#### Dimensions assessed

The definitions and terminology related to food environments that are used in this article are mostly based on the conceptual framework developed by Turner *et al*.^(4)^. We assessed availability (i.e. count, density and proportion) of the different types of food retailers and advertisements, as well as accessibility or physical proximity of schools to the nearest food retailers^(4)^.

#### Food retailers and food advertisements: construct definitions and classification system

Given that there is no consensus on a classification system to categorise food environments as healthy *v*. unhealthy, we opted for a typology derived from the NOVA classification system. The NOVA classification categorises foods into four groups according to the extent of food processing level^(34)^. Foods in group 1 are unprocessed and minimally processed foods such as fresh fruits and vegetables, and flours. Foods in group 2 are processed culinary ingredients such as oils, honey, sugar and salt. Group 3 is for processed foods such as unpackaged breads, canned vegetables and cheeses. Group 4 is for ultra-processed foods such as packaged snacks, chips, chocolates and processed meat. We chose this NOVA-based typology given the evidence that food processing levels, rather than individual nutrients or food items, might be a major driver of childhood obesity with multiple studies associating intakes of ultra-processed foods with overconsumption and increased body weight^(39,40)^. A description of the NOVA-based constructs that we developed and used for this study is given as follows.

##### Food retailers

This included all food or drink establishments within the 800-m buffer zone (including side streets and building complexes) such as eating places, stores, markets, outlets and mobile vendors. Food retailers were first categorised by type into fourteen groups using a checklist adapted for the Tunisian foodscape. This checklist was developed by the research team after extensive discussions among team members; it included definitions, local examples and sample pictures of each type of Tunisian food retailer (see online supplementary material, Supplemental Fig. 1, Additional file 1). The fourteen categories were further grouped into six then three constructs (healthy, mixed, unhealthy) based on the processing level of the prevalent foods sold within the retailer as shown in Table 1. For this, findings of a previous in-store audit conducted in Tunisia were used^(41)^. In the latter study, photos of all food products available in different types of food retailers were taken. A list of 1436 unique varieties of these was established. Four trained nutritionists coded and classified all photographed food products into separate NOVA groups^(34)^. The food retailers were then classified according to the relative abundance of NOVA food groups into unhealthy and healthy retailers.

**Table 1:** Food retailers and advertisements in Greater Tunis: detailed typology and NOVA-based typology

**Food retailers** *		
Detailed typology (14 groups)	NOVA-based typology†	
	Six groups	Three groups
Butcher, poultry and fish stores/markets	Outlets selling mainly unprocessed or minimally processed foods or processed culinary ingredients (> 60 % of foods sold within the retailers are from NOVA groups 1 and 2)	Healthy
Fruit and vegetable stores and markets		
Mobile vendors selling foods from NOVA groups 1 and 2 (e.g. fruits, vegetables)		
Hyper/supermarkets	Hyper/super/mini markets	Mixed *(outlets selling a wide range of products spanning across all four NOVA groups)*
Mini markets *(‘superette’, ‘maghaza*’)		
Corner stores *(‘attar’)*	Corner shops	
Full-service restaurants	Outlets selling mainly unprocessed and processed/ultra-processed foods	
Dairy stores		
Local limited-service restaurants	Limited-service restaurants and retailers	Unhealthy
International fast-food chains		
Mobile vendors selling foods from NOVA groups 3 and 4 (e.g. carbonated beverages, crepes, sandwiches)		
Desserts, fruit cocktails, coffee and tea places	Outlets selling mainly processed or ultra-processed foods (> 60 % of foods sold within the retailers are from NOVA groups 3 and 4)	
Kiosks *(‘kechk’)*		
Bakeries and pastries stores		

Food advertisement sets*,‡		
Healthy: *Advertisement set including solely unprocessed, minimally processed food items or processed culinary ingredients. (Solely NOVA groups 1 and 2)*		
Mixed: *Advertisement set including both unprocessed and processed/ultra-processed food items (all NOVA groups 1–4)*		
Unhealthy: *Advertisement set including solely processed and ultra-processed food items (Solely NOVA groups 3 and 4)*		
Unclear§		

* Food includes beverages.

† Classification of food retailers into the three constructs was based on findings of a previous in-store and in-restaurant audit conducted in Tunisia^(41)^.

‡ All food and beverage advertisements available in one single geographic location (e.g. storefront of a food outlet) were considered as one set of advertisements (i.e. one exposure). Each advertisement set might include several food groups as it can promote more than one food or beverage product.

§ Unclear corresponds to food advertisement sets that could not be categorised because (a) pictures were blurred or (b) it is not possible to deduce the NOVA-processing level^(34)^ of the food items included in the pictures.

##### Outdoor food advertisements

This encompassed all outdoor advertisements promoting food or drink products present within the 800-m road network buffer zone. We included billboards, logos, signs, pictures and storefronts advertisements as well as outdoor pictures or drawings of unbranded food or drink products as these also provide significant food cues. Temporary advertisements, such as those on stationary delivery vehicles, were excluded. For the remaining of this article, the term food advertisements refer to any visual depiction of foods or drinks whether branded or not. All food and beverage advertisements available in one single geographic location (e.g. storefront of a food outlet) were considered as one set of advertisements (i.e. one exposure). Each advertisement set might include several food groups as it can promote more than one food or beverage product. Similar to food retailers, food advertisement sets were grouped into three constructs (healthy, mixed, unhealthy) as shown in Table 1. For this, each food item included within the advertisement set was categorised into the four NOVA groups^(34)^. For comparability purposes, we additionally classified each food item into twenty-one groups using a checklist derived from the WHO nutrient profile model – the latter being a model that categorises foods into permitted and not permitted to be marketed to children^(42, 43)^ (see online supplementary material, Supplemental Table 1, Additional file 1). To avoid any misclassification, a rigorous protocol was implemented whereby two independent researchers reviewed all the geotagged pictures to assign the NOVA and WHO groups. As an example, an advertisement set that included breakfast cereals and apples would receive the following labels: (1) ‘NOVA group 4: breakfast cereals’ and (2) ‘NOVA group 1: fresh fruits and vegetables’. This advertisement set would be further categorised as ‘Mixed: Advertisement set including both unprocessed and processed foods’.

### Covariates

School-level measures including the type of school (private *v*. public) and the departments (i.e. districts) and governorates where schools are located were also collected during fieldwork. Poverty rate (as percentage per capita) and total population count (as total number of individuals) of each department of Greater Tunis were retrieved from a report produced by the National Institute of Statistics in Tunisia, in collaboration with the World Bank^(36)^.

### Data analysis

The geocoded locations of schools, food retailers and food advertisements were visualised using a geographic information system (GIS) software (ArcGIS Pro 3.0.0, ESRI Inc.). Analyses for food retailers and food advertisements were conducted separately

Descriptive analyses were conducted in two ways:

- First, we studied the frequency distribution of types of retailers and advertisement sets pooled over the fifty schools, and this is to provide an overall availability measure (i.e. GIS point data are the unit of analysis).
- Second, we computed the count and density per school. Count was the number of each type of retailer and advertisement set in the 800-m buffer around each school. For schools with overlapping buffers, food retailers and advertisement sets were included in the count of each school. Density was calculated by dividing the count of each type of retailer and advertisement set by the surface area for each school: the surface area was the service area polygon of an 800-m road network buffer (see online supplementary material, Supplemental Fig. 2, Additional file 1). For each school, we also generated the shortest path (proximity) to the closest retailer by type. We used network distance, which accounts for the street network, rather than Euclidean distance as it mimics the actual walking routes^(44)^. Median and inter-quartile range (IQR) across the fifty schools were computed for count, density and proximity data (as data were not normally distributed).

To explore potential factors associated with different types of food retailers or advertisement sets (i.e. healthy, mixed and unhealthy), multinomial regression models with type of retailer or advertisement set as response variables were conducted (using retailer or advertisement set (i.e., GIS data point) as the unit of analysis, respectively). All models accounted for the school-level clustered sample and included the following covariates: type of school (private *v*. public), distance from school to food retailer or advertisement set within each buffer, governorate where school is located, poverty rate and population count of the departments where school is located. Crude and adjusted relative prevalence ratios with 95 % CI and using the ‘healthy’ category as the response reference category were presented.

A sensitivity analysis using 400- and 200-m road network buffer zones was carried out as applying various buffer sizes is recommended to allow comparability across studies^(9)^.

Descriptive geospatial analysis was conducted on ArcGIS Pro version 3.0.0 (ESRI Inc.). All statistical analyses were performed using STATA version 17 (STATA Corporation), and a first type error rate of 0·05 was used.

## Results

Overall, we collected data on 3168 food retailers and 1796 food advertisement sets. As food retailers and advertisement sets available in overlapping buffers were included in the count of each school, we ended up with a total of 3621 retailers and 2098 advertisement sets across the fifty schools. Henceforth, all the analyses presented are based on the latter numbers.

### School neighbourhood food environments

The median counts were 64 (IQR = 47–95) food retailers per school and 36 (IQR = 25–53) food advertisement sets per school (Table 2). Food retailers were more frequent in Tunis, which is the capital and the most urbanised governorate of Tunisia as compared with other governorates (Table 2).

**Table 2:** Availability of food retailers and advertisement sets around fifty primary schools in Greater Tunis

Food* environment within 800 m† of schools	Availability											
	Food retailers‡						Food advertisement sets‡					
	Unit of analysis: GIS point data, *n* 3621		Unit of analysis: School, *n* 50§				Unit of analysis: GIS point data, *n* 2098		Unit of analysis: School, *n* 50§			
	Total count||		Median count per school	IQR	Median density (count/km^2^)¶ per school	IQR	Total count||		Median count per school	IQR	Median density (count/km^2^)¶ per school	IQR
	*n*	%					*n*	%				
Total	3621		64	47–95	59	43–79	2098		36	25–53	33	24–49
Type of food exposure												
Healthy	471	13·0	8	3–14	7	3–12	371	17·7**	6	4–8	6	3–7
Mixed	978	27·0	18	10–28	17	10–22	383	18·3**	5	4–10	5	3–9
Unhealthy	2172	60·0	39	28–52	34	23–45	1255	59·8**	23	13–32	19	12–31
Type of school												
Public	2690	74·3	67	49–95	59	45–81	1538	73·3	41	25–53	34	25–49
Private	931	25·7	59	28–96	48	29–74	560	26·7	35	17–66	27	14–51
Poverty rate of the departments where school is located†† (tertiles)												
Low poverty rate	1449	40·0	56	39–99	58	35–83	815	38·8	28	20–59	26	17–55
Medium poverty rate	1509	41·7	76	55–96	62	51–87	900	42·9	45	29–58	36	29–60
High poverty rate	663	18·3	61	36–77	50	32–66	383	18·3	35	20–43	28	19–38
Total population count of the departments where schools are located†† (quintiles)												
q1	444	12·3	53	39–72	42	38–63	263	12·5	32	16–47	26	17–44
q2	731	20·2	97	50–159	79	45–132	392	18·7	54	27–101	43	25–84
q3	499	13·8	50	24–63	53	32–69	287	13·7	26	11–41	31	14–38
q4	844	23·3	63	47–82	60	45–75	524	25·0	35	25–55	39	24–49
q5	1103	30·5	78	65–99	67	54–81	632	30·1	45	34–53	35	27–58
Governorates where schools are located												
Tunis	1781	49·2	74	55–123	74	51–112	1068	50·9	47	27–85	47	32–61
Ariana	637	17·6	65	63–96	59	46–69	329	15·7	36	32–41	28	26–37
Ben Arous	926	25·6	57	47–83	54	45–81	538	25·6	32	25–52	29	24–49
Manouba	277	7·6	25	10–62	24	14–59	163	7·8	17	5–33	15	6–32

GIS, geographic information system; IQR, inter-quartile range; q, quintile.

* Food including beverages.

† Road network distance in metres.

‡ Retailers that display storefront advertisements were included in the count of both retailers and advertisement sets.

§ Medians and IQR were generated across the fifty schools.

|| Non-standardised counts were generated by summing the GIS data points within the 800-m buffers across the fifty schools. For schools with overlapping buffers, GIS data points were included in the count of each school. Column percentages were computed.

¶ For each of the fifty schools, the 800-m road network buffer yielded a different surface area. The surface area ranged from 0·4 to 1·5 km^2^ with a median of 1·2 km^2^. Density was calculated for each school by dividing the count of retailers or advertisement sets by the surface area of the 800-m buffer (in km^2^).

** Column percentages do not add up to 100 as *n* 89 advertisement sets could not be categorised because (a) pictures were blurred or (b) it is not possible to deduce the NOVA-processing level of the food items included in the pictures^(34)^.

†† Poverty rate (as percentage per capita) and population count (as total number of individuals) of each department of Greater Tunis were retrieved from a report produced by the National Office of Statistics of Tunisia, in collaboration with the World Bank^(36)^. Poverty rates were categorised into tertiles as follows: high poverty rate (7·3–15·2 %); medium poverty rate (4·1–7·1 %) and low poverty rate (0·2–3·8 %). Total population count was categorised into quintiles as follows: q1 (17 408–27 749 individuals); q2 (29 185–40 101); q3 (41 830–57 194); q4 (58 792–84 312) and q5 (86 024–129 693). Each school was matched to its corresponding department’s poverty rate tertile and population quintile.

**Fig. 1:**
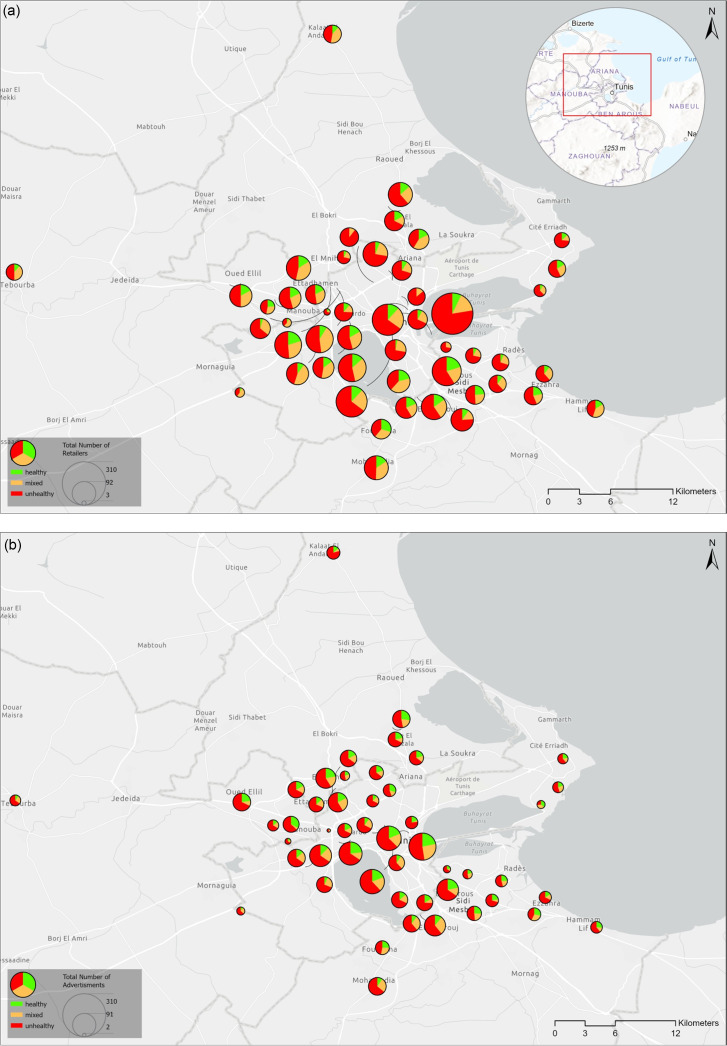
School neighbourhoods in Greater Tunis: Availability of food retailers and food advertisement sets by type. Legend: Each pie represents a school. Availability of healthy, mixed and unhealthy (a) food retailers and (b) food advertisement sets in the Greater Tunis area. The size of the pie reflects the count of food retailers and food advertisement sets. The size of each slice reflects the percentage of total

School neighbourhood food environments in Greater Tunis included predominantly unhealthy retailers and advertisement sets (Fig. 1). According to the NOVA-based typology, around 60 % of food retailers were classified as unhealthy (*n* 2172) and only 13 % were classified as healthy (*n* 471) (Table 2). Similarly, the majority of food advertisement sets included solely ultra-processed foods (around 60 % of all food advertisement sets) while only 18 % included solely unprocessed and minimally processed foods (Table 2). In sensitivity analysis, this predominance of obesogenic food exposures was observed consistently regardless of the buffer size (i.e. 200, 400 and 800 m) (see online supplementary material, Supplemental Table 2, Additional file 1).

### Retail food environment: typology, count, proximity and socio-economic disparities

The most common retailers available within the 800-m buffers were corner stores or ‘attar’ (21·8 %) and desserts/coffee/tea places (20·9 %), followed by kiosks (16·3 %) and limited-service restaurants (16·2 %) (Table 3). Only 6 % of food retailers were fruit and vegetable stores/markets. The limited-service restaurants category predominantly encompassed pizzerias and local sandwich shops (e.g. *mleoui, kabab or chapati sandwich shops)* with less than 1 % consisting of international fast-food chains. While around 22 % of retailers comprised corner stores, hyper/super/mini markets were quite rare around schools (2·1 %). As for proximity, corner stores were the closest to schools (median = 135 m; IQR = 58–215 m) followed by dessert/coffee/tea places (median = 189 m; IQR = 88–295 m) and kiosks (median = 208 m; IQR = 133–293 m) (Table 3).

**Table 3:** The retail food environments around fifty primary schools in Greater Tunis: proximity and availability

Retail food* environment within 800-m† buffer of schools								
			Proximity (m)‡		Availability			
			Unit of analysis: school, *n* 50		Unit of analysis: GIS point data, *n* 3621		Unit of analysis: school, *n* 50	
Typology			Median distance per school||	IQR	Total count¶		Median count per school||	IQR
NOVA-based typology§		Detailed typology			*n*	%		
Healthy	Outlets selling mainly UNP foods	Butcher, poultry and fish stores/markets	304	155–440	243	6·7	4	2–8
		Fruit and vegetable stores and markets	291	200–419	216	6·0	3·5	2–6
		Mobile vendors**	–		12	0·3	0	
Mixed	Hyper/super/mini markets	Hyper/Supermarkets	409	265–554	77	2·1	1	1–2
		Mini markets *(‘superette’, ‘maghaza*’)	400	227–641	35	1·0	0	0–1
	Corner stores	Corner stores *(‘attar’)*	135	58–215	788	21·8	14	6–24
	Outlets selling both UNP and P foods	Full-service restaurants	435	305–685	47	1·3	0	0–1
		Dairy stores	294	145–481	31	0·9	0	0–1
Unhealthy	Limited-service restaurants and retailers	Local limited-service restaurants	217	150–325	586	16·2	9	5–13
		International fast-food chains	537	369–695	3	0·1	0	
		Mobile vendors**	–		17	0·5	0	
	Outlets selling mainly P foods	Desserts, fruit cocktails, coffee and tea places	189	88–295	755	20·9	12	9–19
		Kiosks *(‘kechk’)*	208	133–293	590	16·3	12	7–15
		Bakeries and pastries stores	339	192–496	221	6·1	4	2–6

GIS, geographic information system; IQR, inter-quartile range; P, processed food; UNP, unprocessed food.

* Food including beverages.

† Road network distance in metres.

‡ For each of the fifty schools, road network distance (in metres) from the school to the nearest retailer, by type, was generated. Median and IQR across the fifty schools are presented in the table.

§ NOVA classification^(34)^. In this table, unprocessed foods refer to unprocessed/minimally processed foods and processed culinary ingredients (NOVA groups 1 and 2). Processed foods refer to processed and ultra-processed foods (NOVA groups 3 and 4).

|| Medians and IQR were generated across the fifty schools.

¶ Non-standardised counts were generated by summing the GIS data points within the 800-m buffers across the fifty schools. For schools with overlapping buffers, GIS data points were included in the count of each school. Column percentages were computed.

** Mobile vendors include (a) vendors selling unprocessed/minimally processed foods such as vegetables, fruits and popcorn and (b) vendors selling processed/ultra-processed foods such as sandwiches, carbonated beverages and crepes. The median distance from schools to nearest mobile vendors was not generated.

Table 4 explores the association between school characteristics and types of food retailers. Analyses were performed with the GIS point data being the unit of analysis. The adjusted relative prevalence ratio of unhealthy to healthy food retailers was 1·9 times significantly higher in schools located in the richest areas (i.e. lowest poverty rates) as compared with the poorest areas (adjusted relative prevalence ratio = 1·9(1·3–2·7), *P*-value = 0·001). The same analysis was performed in the six NOVA-based categories of food retailers to explore the difference in sub-categories (see online supplementary material, Supplemental Table 3, Additional file 1). Apart from corner stores, the adjusted relative prevalence ratios for all the remaining types of food retailers were significantly higher around schools located in the richest areas as compared with the poorest ones in the adjusted models (with the reference outcome being outlets selling mainly unprocessed foods). For corner stores, the opposite pattern was observed but without reaching statistical significance.

**Table 4:** Association between type of food retailers and school characteristics across primary schools in Greater Tunis

Retail food† environment within 800 m‡ of schools	Unit of analysis: GIS point data§						Multinomial regression (reference category: healthy) ||							
	Healthy (*n* 471)		Mixed (*n* 978)		Unhealthy (*n* 2172)		Mixed				Unhealthy			
	*n*	%	*n*	%	*n*	%	RPR	95 % CI	ARPR¶	95 % CI	RPR	95 % CI	ARPR¶	95 % CI
Type of school														
Public	337	71·5	752	76·9	1601	73·7	ref		ref		ref		ref	
Private	134	28·5	226	23·1	571	26·3	0·8	0·6, 1·0	**0·7**	**0·5, 0·9** **	1·0	0·7, 1·4	0·9	0·7, 1·4
Distance from school to food retailers within buffers (m)††														
≤ 200	52	11·0	109	11·1	201	9·3	ref		ref		ref		ref	
> 200 to ≤ 400	107	22·7	244	24·9	479	22·1	1·1	0·7, 1·7	1·1	0·7, 1·7	1·2	0·8, 2·0	1·3	0·9, 2·0
> 400 to ≤ 800	312	66·2	625	63·9	1492	68·7	1·0	0·7, 1·7	1·0	0·7, 1·6	1·4	0·9, 2·1	**1·5**	**1·0, 2·1** *
Poverty rate of the departments where schools are located‡‡ (tertiles)														
High poverty rate	113	24·0	223	22·8	327	15·1	ref		ref		ref		ref	
Medium poverty rate	197	41·8	423	43·3	889	40·9	1·0	0·7, 1·5	1·0	0·8, 1·4	**1·5**	**1·1, 2·1** *	1·2	0·8, 1·8
Low poverty rate	161	34·2	332	33·9	956	44·0	1·1	0·8, 1·5	1·2	0·9, 1·5	**2·2**	**1·5, 3·3** ***	**1·9**	**1·3, 2·7** **
Total population count of the departments where schools are located‡‡ (quintiles)														
q1	41	8·7	121	12·4	282	13·0	ref		ref		ref		ref	
q2	84	17·8	141	14·4	506	23·3	**0·5**	**0·3, 0·9** *	**0·5**	**0·3, 0·9** *	0·9	0·4, 1·9	0·9	0·5, 1·7
q3	50	10·6	131	13·4	318	14·6	0·9	0·5, 1·6	1·1	0·7, 1·9	1·0	0·5, 2·0	1·2	0·5, 2·6
q4	147	31·2	249	25·5	448	20·6	**0·6**	**0·3, 0·9** *	0·7	0·4, 1·1	**0·4**	**0·2, 0·7** **	**0·5**	**0·3, 0·9** *
q5	149	31·6	336	34·4	618	28·5	0·7	0·5, 1·2	0·9	0·5, 1·5	0·6	0·3, 1·1	0·6	0·3, 1·1
Governorates where schools are located														
Tunis	194	41·2	468	47·9	1119	51·5	ref		ref		ref		ref	
Ariana	83	17·6	181	18·5	373	17·2	0·9	0·7, 1·2	0·9	0·6, 1·2	0·8	0·5, 1·2	1·1	0·8, 1·7
Ben Arous	150	31·8	235	24·0	541	24·9	**0·7**	**0·5, 0·9** *	**0·8**	**0·6, 1·0** *	0·6	0·4, 1·0	0·8	0·6, 1·1
Manouba	44	9·3	94	9·6	139	6·4	0·9	0·6, 1·3	1·0	0·7, 1·4	**0·5**	**0·4, 0·8** **	1·0	0·6, 1·6

ARPR, adjusted relative prevalence ratio; GIS, geographic information system; IQR, inter-quartile range; q, quintile; ref, reference category; RPR, relative prevalence ratio.

Numbers in bold indicate statistical significance:

* *P* < 0·05.

** *P* < 0·01.

*** *P* < 0·001.

† Food including beverages.

‡ Road network distance in metres.

§ Non-standardised counts were generated by summing the GIS data points within the 800-m buffers across the fifty schools. For schools with overlapping buffers, GIS data points were included in the count of each school. Column percentages were computed.

|| Multinomial regressions were conducted with the reference category being ‘Healthy’ food retailers.

¶ Models adjusted for all the variables presented in column one (i.e. type of school, poverty rates and total population count of the areas where schools are located, governorates where schools are located and distance from school to food exposures).

†† Distance (road network) in metres from school to food retailers within each buffer.

‡‡ Poverty rate (as percentage per capita) and population count (as total number of individuals) of each department of Greater Tunis were retrieved from a report produced by the National Office of Statistics of Tunisia, in collaboration with the World Bank^(36)^. Poverty rates were categorised into tertiles as follows: high poverty rate (7·3–15·2 %); medium poverty rate (4·1–7·1 %) and low poverty rate (0·2–3·8 %). Total population count was categorised into quintiles as follows: q1 (17 408–27 749 individuals); q2 (29 185–40 101); q3 (41 830–57 194); q4 (58 792–84 312) and q5 (86 024–129 693). Each school was matched to its corresponding department’s poverty rate tertile and population quintile.

### Food advertisements: typology, count and socio-economic disparities

Only 1 % of advertisement sets consisted of billboards (see online supplementary material, Supplemental Table 4, Additional file 1). The remaining sets were located on storefronts and store signs of shops – mostly on corner stores (28 % of all food advertisement sets) and kiosks (23 % of all food advertisement sets) – and were predominantly promoting unhealthy food products (see online supplementary material, Supplemental Table 4, Additional file 1).

**Fig. 2:**
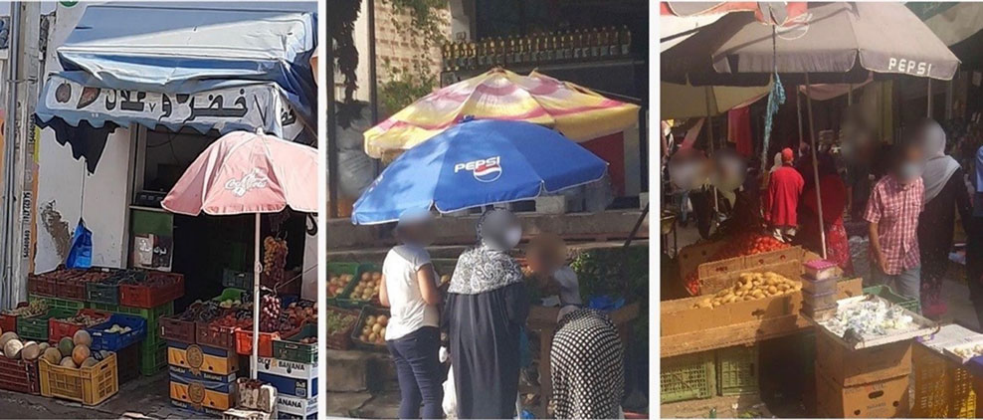
Sample pictures of fruit and vegetable stores/markets with parasols promoting ultra-processed foods. Legend: Pictures were taken by data collectors whose names are mentioned in the Acknowledgements section. Permission to use their pictures was granted

Advertisement sets present on fruit and vegetable stores/markets were mostly promoting solely unprocessed or minimally processed foods – although around 30 % of these sets included ultra-processed food products. The latter consisted of promotional parasols for carbonated and sugar-sweetened beverages, which were used by vendors to protect their fruits and vegetables from the sun (Fig. 2). A substantial number of store signs were also promotional products for a dairy brand. For billboards, around 86 % included solely processed and/or ultra-processed foods (see online supplementary material, Supplemental Table 4, Additional file 1).

As shown in Table 5, the 2098 food advertisement sets included 3622 different food groups as one food advertisement set might include several products. The prevailing food group promoted was carbonated beverages and sugar-sweetened beverages (22 %); this was followed by sweet snacks (9·4 % and 7·2 %). Around 11 % consisted of non-sweetened beverages and only 3 % of fresh fruits and vegetables.

**Table 5:** Food groups promoted around primary schools in Greater Tunis, by type and distance from school

Food group type*	Count across the fifty schools†							
	≤ 200 m‡		200–400 m‡		400–800 m‡		Total	
	*n*	%	*n*	%	*n*	%	*n*	%
NOVA groups 3 and 4§								
Carbonated beverages and sugar-sweetened beverages||	82	19·9	185	23·0	529	22·0	796	22·0
Cakes, sweet biscuits and pastries, and other sweet bakery products||	48	11·7	77	9·6	216	9·0	341	9·4
Chocolate and sugar confectionery, energy bars, sweet topping, ice cream and sorbets||	23	5·6	55	6·8	183	7·6	261	7·2
Flavoured yogurt, sweetened milk and dairy products	47	11·4	87	10·8	245	10·2	379	10·5
Food restaurant items (dishes and sandwiches), ready-made and convenience foods and composite dishes	27	6·6	59	7·3	204	8·5	290	8·0
Savoury/salty snacks (including salted nuts)	13	3·2	27	3·3	77	3·2	117	3·2
Processed meat, poultry and similar||	8	1·9	18	2·2	55	2·3	81	2·2
Bread, bread types and breakfast cereals	2	0·5	8	1·0	42	1·7	52	1·4
Miscellaneous including canned fish, processed fruits and vegetables, and processed sauces and dressings	8	2·1	7	0·9	28	1·2	43	1·3
NOVA groups 1 and 2§								
Non-sweetened beverages (water, coffee, tea, etc.)	43	10·4	100	12·4	247	10·3	390	10·8
Fresh and frozen meat, poultry, fish and eggs	21	5·1	38	4·7	126	5·2	185	5·1
Fresh and frozen fruits and vegetables	11	2·7	16	2·0	76	3·2	103	2·8
Fresh, dried or cooked pasta, rice and grains	7	1·7	9	1·1	41	1·7	57	1·6
Milk and unflavoured yogurt	6	1·5	11	1·4	36	1·5	53	1·5
Butter, and other fats and oils	4	1·0	4	0·5	21	0·9	29	0·8
Miscellaneous including honey, spices and herbs	6	1·5	10	1·2	23	1·0	39	1·1
Unclear¶	56	13·6	95	11·8	255	10·6	406	11·2
Total number of promoted food groups	412		806		2404		3622	
Total number of food advertisement sets**	220		489		1389		2098	

* Food including beverages.

† Non-standardised counts were generated by summing food groups promoted (and not food advertisement sets) within the 800-m buffers and across the fifty schools, by type and distance from school. For schools with overlapping buffers, food groups were included in the count of each school. Column percentages of total number of promoted food groups were computed.

‡ Distance (road network) in metres from school to advertisement sets within each buffer.

§ NOVA classification^(34)^.

|| Food groups for which marketing is prohibited or not permitted to children based on the nutrient profile model in the WHO Eastern Mediterranean Region^(43)^.

¶ Unclear corresponds to food items that could not be categorised because (a) pictures were blurred or (b) it is not possible to deduce the NOVA-processing level of the food items included in the pictures^(34)^.

** All food and beverage advertisements available in one single geographic location (e.g. storefront of a food outlet) were considered as one set of advertisements. Each advertisement set might include several food groups because it is promoting more than one food or beverage product.

Distributions of the three NOVA-based types of food advertisement sets (i.e. healthy, mixed and unhealthy) did not significantly differ by distance from school nor by school characteristics (see online supplementary material, Supplemental Table 5, Additional file 1).

## Discussion

This study pertained to the Greater Tunis area, typical of a highly developed and urbanised area in the MENA region with an ongoing nutrition transition and increasing rates of childhood obesity. We studied the built food environment around primary schools using geospatial methods and a typology of food retailers and advertisements derived from the NOVA classification^(34)^. School neighbourhood food environments included predominantly unhealthy food retailers and advertisements. Obesogenic food retailers were more prevalent around schools located in the richest areas.

This study contributes to the scarce body of evidence on objectively measured food environments in LMIC. To the best of our knowledge, this is the first study to assess built food environments – specifically the availability of food retailers and advertisements around schools – in an Arab country using geospatial static methods.

### Predominance of unhealthy food exposures

School neighbourhoods included a substantial number of food retailers and advertisements; the majority of which were classified as unhealthy.

The most common food retailers consisted of small traditional shops including corner stores (*‘attar*’) and kiosks. The count of fast-food restaurants in school neighbourhoods was higher than figures reported in Hong Kong^(45)^, Mexico^(46)^ and Berkshire, United Kingdom^(47)^ but lower than those reported in New York City^(48)^. Yet, interpretation should be done with caution since the construct ‘fast-food restaurants’ might be defined differently across these studies.

As for food advertisements, the vast majority were promoting ultra-processed and high energy-dense foods, including sweet snacks as well as carbonated and sugar-sweetened beverages, a finding which corroborates prior studies from other countries^(18,49)^. Storefronts of corner stores and kiosks also included an overabundance of unhealthy food products located in one geographic place. This predominance of obesogenic food exposures is further exacerbated by the fact that we found healthy food retailers – such as fruit and vegetable markets – to be infiltrated by unhealthy promotional products, underlining the need to protect these sparse healthy spaces from unhealthy food marketing.

### Disparities in food environments by school neighbourhood socio-economic status

Unhealthy food retailers were more prevalent around schools located in the richest areas as compared with the poorest ones. This is consistent with early stage four of the nutrition transition model, which posits that availability of unhealthy, processed and high energy-dense foods – which contributes to obesogenic environments – increases as income rises^(32)^. However, it is well-acknowledged that a wealth-gradient exists for overweight and obesity with evidence showing that overweight/obesity burdens – and thus obesogenic food environments – shift from wealthier to poorer sub-populations as a country develops^(50)^. We can conjecture that this shift has not occurred yet in Greater Tunis, which explains why our finding contradicts reports from HIC^(21,22,51)^ where unhealthy food environments tend to prevail in socio-economically deprived areas.

### Challenges in assessing food environments in a low- and middle-income country

Our study adds to the body of knowledge on the challenges encountered when assessing food environments in LMIC. The main challenge pertained to the lack of valid, standardised and ‘cross-context equivalent’ metrics – as described by others^(28)^.

Indeed, most of the literature on food retailers uses constructs such as ‘fast-food restaurants’, ‘supermarkets’, ‘grocery stores’ or ‘convenience stores’. However, these constructs are difficult to apply to traditional Tunisian retailers. For example, traditional corner stores or *‘attar’* are often labelled as convenience stores despite offering a relatively high proportion of healthy food options. This is why we developed a checklist adapted to the Tunisian foodscape.

Another challenge – albeit not specific to LMIC – pertained to the multiple definitions and scopes available in the literature for food retailer constructs, which hinder comparability among studies. In their article, Wilkins *et al*.^(9)^ divided the constructs of ‘fast-food restaurants’, ‘supermarkets’ and ‘convenience stores’ into narrow, moderate and broad scopes^(9)^. Our data showed that the frequency of the construct ‘supermarkets’ changed from 2 % to 25 % (a 10-fold increase) when using the moderate *v*. broad scopes (i.e. if we include corner stores within the ‘supermarkets’ construct) (see online supplementary material, Supplemental Fig. 3, Additional file 1).

Apart from classifying food retailers into constructs, the lack of consensus on one classification system or index to categorise these constructs as healthy or unhealthy compelled us to adapt a NOVA-based classification system. Despite current debates surrounding the NOVA classification’s lack of clear guidelines on how to classify foods based on ingredients^(52)^, we opted for this system given the available evidence linking ultra-processed foods to adverse nutritional outcomes^(40)^. For food advertisements, we followed a thorough protocol to avoid any misclassification, whereby two independent researchers reviewed all the pictures and assigned food items into one of the four NOVA categories. Besides the intense logistics required to undertake a ground-truthing study, security concerns emerged during fieldwork. These largely related to the perception by food vendors (particularly informal vendors) that GIS mapping and pictures of their stores could negatively affect their business and lead to, for example, shop closure, control from municipalities and policy action.

### Strengths and limitations

Our study has several strengths. First, it is a representative study (through the sampling approach) of primary schools in Greater Tunis and therefore gives a solid description of the status-quo of school food environments of this middle-income Arab city. Additionally, an in-person mapping using Global Positioning System techniques along with a thorough protocol and rigorous training of fieldworkers was conducted to ensure high quality data collection. We collected data on all types of food retailers and did not restrict our research to fast-food restaurants or grocery stores; we also simultaneously collected data on food advertisements. We described our data using several metrics (count, proportion, density, proximity) and buffer sizes (200-, 400- and 800-m road network buffers) to facilitate comparison across studies. Also, given that this is the first study to assess food environment in Greater Tunis using geospatial methods, the data that we generated can be used as a baseline data for future monitoring studies as well as in future research looking at associations between school neighbourhood food environments and children’s nutritional outcomes. Our study will also contribute to identifying policy and programme levers for intervention, with the potential to improve children’s nutritional status in Tunisia and countries with similar context. Our research also includes some limitations. The main one pertains to the multitude of definitions and methods used by researchers to classify food retailers into types and/or constructs (e.g. healthy/unhealthy). This compelled us to develop our own NOVA-based typology, which hinders comparability across studies. Additionally, food retailers were classified as healthy or unhealthy based on an in-store audit conducted on a subsample of retailers, which might lead to some misclassification bias. In-store audits might be essential to assertively assign a healthy or unhealthy label to retailers. Yet, they are costly, time consuming and difficult to conduct systematically on all retailers. We also only described school neighbourhood food environments and did not include food environments of other places visited by children such as inside schools, home or home neighbourhoods. While GIS mapping and in-store audits give us information on the quality of the external or built food environments, they should be complemented with qualitative interviews to explore how children’s food choices are influenced by the density and types of food retailers and food advertisements. Finally, our study was conducted in the midst of the COVID-19 pandemic which caused significant disruptions to the global food system, including changes in food supply chain and consumer eating behaviours^(53,54)^ – all of this might have altered the Tunisian foodscape to some extent^53,54^.

### Future recommendations and policy implications

Food environments are one of the many entry points into food systems for improving children’s dietary intakes. Intervening at the level of food environments might be more effective in modifying children’s diets than individual behavioural interventions that had limited success^(55)^. This is all the more necessary since findings from the larger SCALE study^(35)^ – of which the present study is part – revealed that about three quarters of children living in Greater Tunis tend to walk to school, and around 30 % of them purchased food from stores available on their way to/from schools in the 24 hours prior to survey administration (unpublished data from SCALE study). Considering the density of obesogenic food retailers and advertising in school neighbourhoods, strategies used in other contexts and for other harmful behaviours, such as tobacco use^(56,57)^, could inform the development of effective interventions to enable healthy environments around schools in Greater Tunis. Our results call for regulation of the urban zoning area around schools to monitor and reduce the density of unhealthy food retailers and food advertisements in the vicinity of schools. As we expect the wealth-gradient shift to unfold in Tunisia, policies should be put in action to protect schoolchildren from low socio-economic status neighbourhoods from the expected proliferation of obesogenic food exposures. The number and type of advertisements on storefronts should be limited, especially those on corner stores and kiosks. Promotional products for ultra-processed foods should not be allowed to be used in healthy food spaces or as store signs. Similar to the one implemented in the United Kingdom^(58)^, a law prohibiting advertisements within close distance of schools, such as 200 m, might be considered – although the efficacy of such measures has not yet been demonstrated. The quality and type of food products sold by food retailers around schools should also be controlled so that ultra-processed food products do not exceed a pre-defined threshold. Mandatory food labelling and warning labels for food items might be an additional strategy to limit demand for unhealthy products. The influence of school neighbourhood food environments on children’s purchasing behaviours and diets in this context should be further explored.

## Conclusions

Our study collected monitoring data on the built (i.e. external) food environments in Greater Tunis and adds to the body of knowledge on the challenges encountered when assessing food environments in an LMIC. Overall, school neighbourhood food environments in Greater Tunis included predominantly unhealthy food retailers and food advertisements underlining the need to promote healthy environments around Tunisian schools. On the other hand, unhealthy food retailers were more prevalent around schools located in the richest areas – which was not unexpected in this nutrition transition setting. Mapping of LMIC food environments is crucial to document the impact of these nutrition transitions on children’s dietary intake and weight status. Therefore, our next step will be to study the association between school neighbourhood food environments and children’s nutritional status. This will help in identifying policy and programme levers for intervention to improve children’s diets and lessen the burden of obesity in Tunisia and countries with similar contexts.

## Supporting information

Akl et al. supplementary material Akl et al. supplementary material

## Supplementary material

For supplementary material accompanying this paper visit https://doi.org/10.1017/S1368980023002860
