# Supplementary material for: Urban school neighbourhoods dominated by unhealthy food retailers and advertisements in Greater Tunis: a geospatial study in the midst of the nutrition transition
Source: Public Health Nutr. 2024 Jan 3;27(1):e44. doi: 10.1017/S1368980023002860 (PMC10882541; doi:10.1017/S1368980023002860)
Supplement: Akl et al. supplementary material [file S1368980023002860sup001.docx]

Additional file 1

Supplementary materials

[**Supplementary figure 1. Tunisian foodscape: typology with sample pictures.** 2](#_Toc127946923)

[**Supplementary table 1. Food advertisements typology.** 4](#_Toc127946924)

[**Supplementary table 2. Availability of food retailers and advertisement-sets around primary schools in Greater Tunis by buffer size.** 5](#_Toc127946925)

[**Supplementary table 3**. **Association between type of food retailers and school characteristics across primary schools in Greater Tunis.** 6](#_Toc127946926)

[**Supplementary table 4. Frequency and location of food advertisement sets around 50 primary schools in Greater Tunis**. 8](#_Toc127946927)

[**Supplementary table 5. Associations between type of food advertisement-sets and school characteristics across primary schools in Greater Tunis.** 9](#_Toc127946928)

[**Supplementary figure 2. Sample map of an 800-meter road network buffer around one school (service area polygon).** 11](#_Toc127946929)

[**Supplementary figure 3**. **The retail food environments in Greater Tunis using different construct definitions.** 12](#_Toc127946930)

[**References** 13](#_Toc127946931)

# **Supplementary figure 1. Tunisian foodscape: typology with sample pictures.**


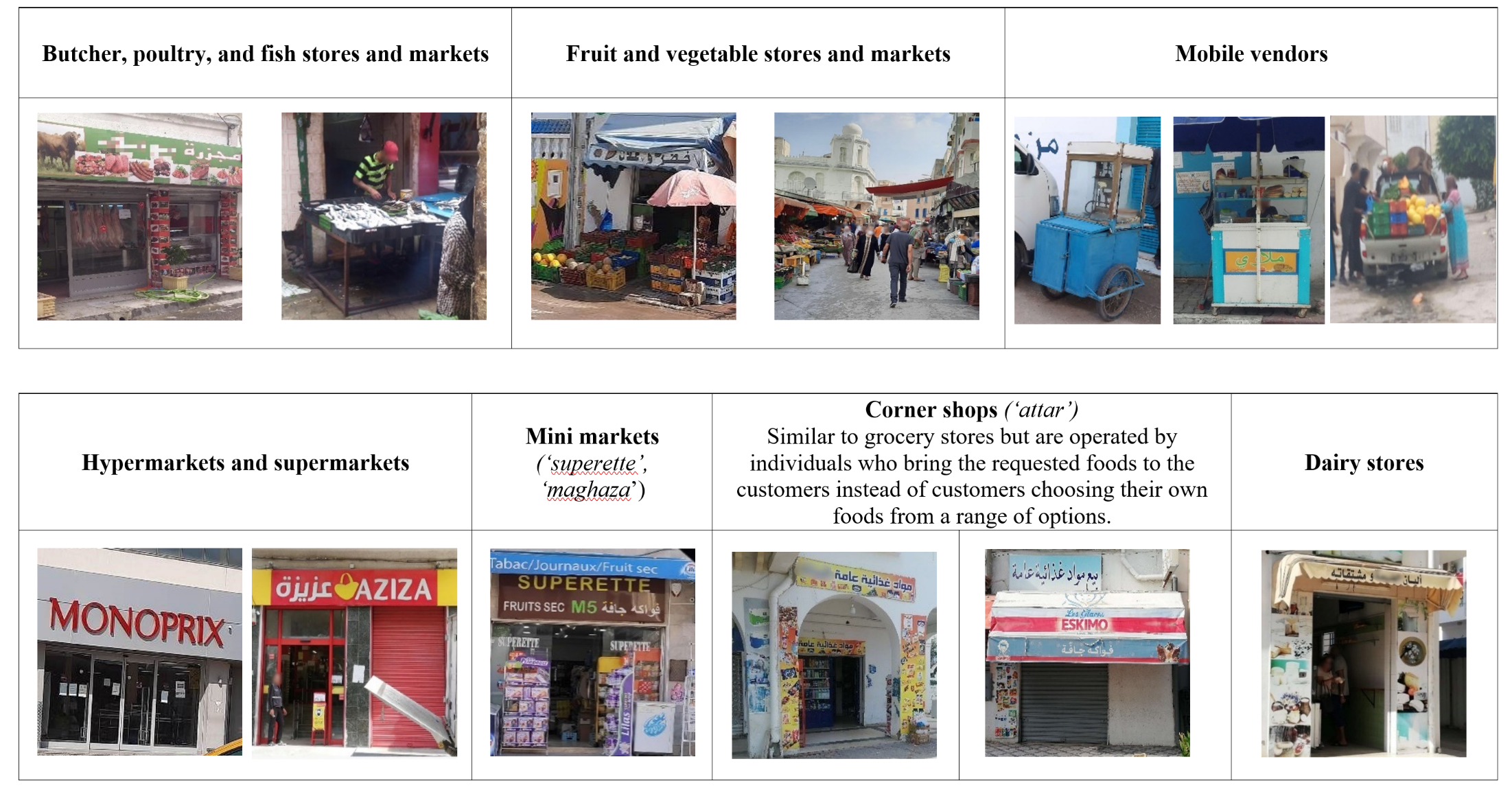


**
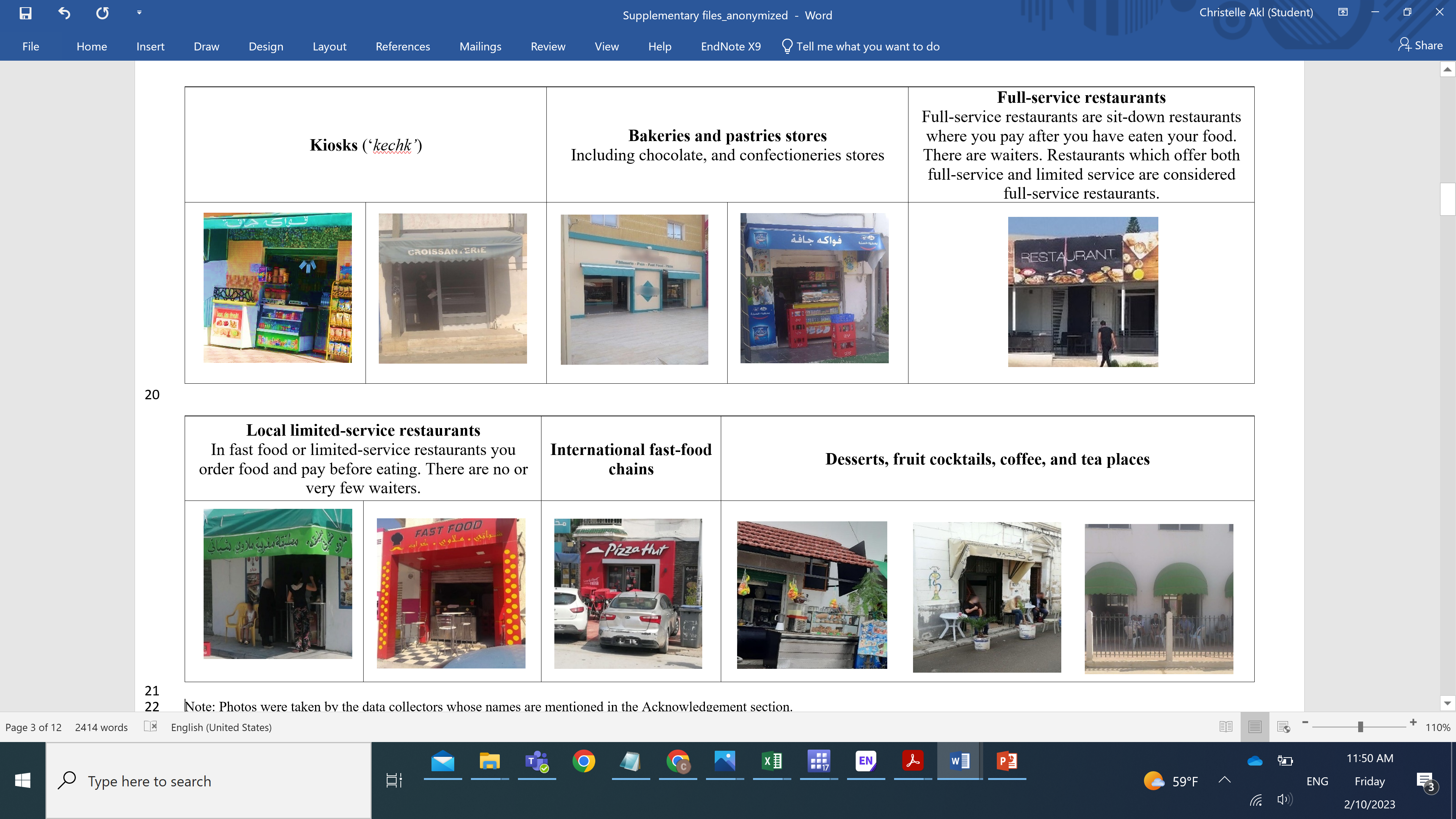
**

Note: Pictures were taken by data collectors whose names are mentioned in the Acknowledgement section.

# **Supplementary table 1. Food advertisements typology.**

| **Food item** ^a^ | |
| --- | --- |
| **Detailed typology**  **(21 groups)** | **NOVA-based classification** ^b^ |
|  |  |
| Fresh and frozen fruits and vegetables | **Healthy:**  *Solely NOVA groups 1 and 2* |
| Fresh, dried or cooked pasta, rice and grains |  |
| Milk and unflavored yoghurt |  |
| Fresh and frozen meat, poultry, fish, and eggs |  |
| Non-sweetened beverages (water, coffee, tea, etc.) |  |
| Butter, and other fats and oils |  |
| Sugar, honey |  |
| Spice and herbs belonging to NOVA groups 1 or 2 |  |
| Canned or processed fruits and vegetables | **Unhealthy:**  *Solely NOVA groups 3 and 4* |
| Bread and bread types |  |
| Breakfast cereals |  |
| Flavored yoghurt, sweetened milk, and dairy products |  |
| Processed meat, poultry, and similar |  |
| Canned fish, and sauces and dressings. |  |
| Cakes, sweet biscuits and pastries, and other sweet bakery products |  |
| Chocolate and sugar confectionery, energy bars, sweet topping, ice cream, and sorbets |  |
| Savory/salty snacks (including salted nuts) |  |
| Carbonated beverages and sugar-sweetened beverages |  |
| Food menu/restaurant items (dishes and sandwiches), ready-made and convenience foods and composite dishes (including ready-to-eat soup) |  |
| Sauces and dressings belonging to NOVA groups 3 or 4 |  |
| **Unclear** ^c^ | |

^a^ Food including beverages.

^b^ NOVA classification ^(1)^. Advertisements were also categorized as healthy, unhealthy, or mixed. Advertisements including both unprocessed and processed/ultra-processed foods (i.e., all four NOVA groups) were labeled as mixed.
^c^ Unclear corresponds to food items that could not be categorized because (a) pictures were blurred or (b) it is not possible to deduce the NOVA-processing level of the food items included in the pictures ^(1)^.

# **Supplementary table 2. Availability of food retailers and advertisement-sets around primary schools in Greater Tunis by buffer size.**

| **Food environment ^a^** | **Buffer size ^b^** | | | | | |
| --- | --- | --- | --- | --- | --- | --- |
|  | **800m** | **400m** | **200m** | **800m** | **400m** | **200m** |
|  | **Unit of analysis: School, n=50** | | | **Unit of analysis: GIS point data** | | |
|  | **Median count (IQR) per school ^c^** | | | **n ^d^** | | |
|  |  | | | **n=3621** | **n=1192** | **n=362** |
| **Food retailers** **^e^** |  |  |  |  |  |  |
| Healthy | 8 (3-14) | 2 (1-5) | 0 (0-1) | 471 | 159 | 52 |
| Mixed | 18 (10-28) | 6 (4-9) | 2 (1-4) | 978 | 353 | 109 |
| Unhealthy | 39 (28-52) | 11 (6-18) | 3 (1-6) | 2172 | 680 | 201 |
| **Food advertisement sets ^e^** |  |  |  |  |  |  |
| Healthy | 6 (4-8) | 2 (1-4) | 0 (0-1) | 371 | 113 | 40 |
| Mixed | 5 (4-10) | 2 (1-4) | 0 (0-1) | 383 | 127 | 47 |
| Unhealthy | 23 (13-32) | 7 (4-11) | 2 (0-4) | 1255 | 415 | 123 |
| Unclear ^f^ | 1 (0-3) | 0 (0-1) | 0 | 89 | 34 | 10 |

Note. GIS: Geographic Information System; IQR: interquartile range; m: meters; q: quintile.

^a^ Food including beverages.

^b^ Buffer size based on road-network distances from schools in meters.

^c^ Medians and IQRs were generated across the 50 schools.

^d^ Non-standardized counts were generated by summing the GIS data points within the buffer zones across the 50 schools. For schools with overlapping buffers, GIS data points were included in the count of each school.

^e^ Retailers that display storefront advertisements were included in the count of both food retailers and food advertisement sets.

^f^ Unclear corresponds to food advertisement-sets that could not be categorized because (a) pictures were blurred or (b) it is not possible to deduce the NOVA-processing level of the food items included in the pictures ^(1)^.

# **Supplementary table 3**. **Association between type of food retailers and school characteristics across primary schools in Greater Tunis.**

|  | **Retail food** ^a^ **environment within 800 m** ^b^ **of schools** | | | | | | | | | | | |
| --- | --- | --- | --- | --- | --- | --- | --- | --- | --- | --- | --- | --- |
|  | Multinomial regression ^c^:  (Reference category: Outlets selling mainly UNP foods) | | | | | | | | | | | |
|  | **Hyper/super/mini markets** | | **Corner shops** | | **Outlets selling mainly UNP & P foods** | | **Limited-service food restaurants and retailers** | | | **Outlets selling mainly**  **P foods** | | |
|  |  | | | | | | | | | | | |
|  | RPR  [95% CI] | ARPR ^d^  [95% CI] | RPR  [95% CI] | ARPR ^d^  [95% CI] | RPR  [95% CI] | ARPR ^d^  [95% CI] | RPR  [95% CI] | | ARPR ^d^  [95% CI] | RPR  [95% CI] | | ARPR ^d^  [95% CI] |
| **Type of school** | | | | | | | | | | | | |
| Public | ref | ref | ref | ref | ref | ref | ref | | ref | ref | | ref |
| Private | 1.1[0.7-1.7] | 0.8[0.5-1.3] | **0.7*[0.5-1.0]** | **0.6**[0.5-0.8]** | 1.5[0.6-4.0] | 1.4[0.7-2.8] | 0.9[0.6-1.4] | | 0.9[0.6-1.4] | 1.0[0.7-1.4] | | 1.0[0.7-1.4] |
| **Poverty rate of the departments where school are located ^e^ (tertiles)** |  | | |  |  | | |  | | |  | |
| High poverty rate | ref | ref | ref | ref | ref | ref | ref | | ref | ref | | ref |
| Medium poverty rate | 1.6[0.8-3.1] | 1.7[0.8-3.8] | 1.0[0.7-1.4] | 1.0[0.7-1.4] | **3.9*[1.3-11.5]** | 3.4[1.0-11.7] | **2.4***[1.6-3.6]** | | **2.0**[1.3-3.2]** | 1.3[0.9-1.8] | | 1.1[0.7-1.6] |
| Low poverty rate | **2.2*[1.1-4.4]** | **3.4**[1.4-7.9]** | 0.8[0.6-1.1] | 0.9[0.6-1.2] | **14.0***[4.5-44.0]** | 12.4[3.9-39.0] | **3.6***[2.3-5.8]** | | **3.0***[1.8-4.8]** | **1.9**[1.3-2.9]** | | **1.7**[1.2-2.4]** |
| **Total population count of the departments where schools are located ^e^ (quintiles)** |  | | |  |  | | |  | | |  | |
| q1 | ref | ref | ref | ref | ref | ref | ref | | ref | ref | | ref |
| q2 | 0.6[0.3-1.2] | **0.5*[0.3-1.0]** | **0.4**[0.2-0.7]** | **0.5**[0.3-0.8]** | 2.7[0.8-9.0] | **2.5* [1.0-6.0]** | 0.9[0.4-2.1] | | 0.9[0.5-1.8] | 0.8[0.4-1.8] | | 0.9[0.4-1.6] |
| q3 | 1.7[0.8-3.2] | 1.8[1.0-3.5] | 0.7[0.4-1.4] | 0.9[0.5-1.6] | 3.0[1.0-9.2] | **3.7*[1.2-11.2]** | 0.8[0.4-1.7] | | 1.0[0.5-2.3] | 1.1[0.5-2.2] | | 1.2[0.6-2.7] |
| q4 | 0.5[0.3-1.1] | 0.6[0.3-1.1] | **0.6*[0.3-1.0]** | 0.7[0.4-1.2] | 0.6[0.2-1.4] | 0.7[0.2-2.4] | **0.3***[0.2-0.6]** | | **0.4*[0.2-0.8]** | **0.5**[0.3-0.8]** | | 0.5[0.3-1.0] |
| q5 | 0.7[0.3-1.6] | 0.5[0.2-1.1] | 0.7[0.4-1.2] | 0.9[0.5-1.6] | 1.8[0.6-5.1] | 1.0[0.3-2.7] | 0.6[0.3-1.2] | | 0.6[0.3-1.1] | 0.6[0.4-1.1] | | 0.6[0.3-1.1] |
| **Governorates where schools are located** |  | | |  |  | | |  | | |  | |
| Tunis | ref | ref | ref | ref | ref | ref | ref | | ref | ref | | ref |
| Ariana | 1.3[0.6-3.0] | **2.5*[1.2-5.4]** | 0.9[0.6-1.2] | 0.7[0.5-1.0] | 0.9[0.3-3.2] | **3.0*[1.2-7.8]** | 0.7[0.4-1.3] | | 1.3[0.8-2.1] | 0.8[0.5-1.2] | | 1.1[0.8-1.6] |
| Ben Arous | 1.2[0.7-2.0] | **1.6*[1.0-3.5]** | **0.7*[0.5-0.9]** | **0.7*[0.5-1.0]** | **0.4*[0.2-0.9]** | 0.7[0.3-1.3] | **0.4**[0.3-0.8]** | | 0.6[0.4-0.8] | 0.7[0.4-1.1] | | 0.9[0.6-1.2] |
| Manouba | 0.9[0.4-2.0] | 1.8[0.8-3.8] | 1.0[0.6-1.5] | 0.9[0.6-1.4] | **0.2*[0.04-0.9]** | 1.0[0.3-3.1] | **0.3***[0.2-0.5]** | | 0.9[0.5-1.8] | **0.6*[0.4-0.9]** | | 1.0[0.6-1.6] |
| **Distance from school to food exposure (m) ^f^** |  |  |  |  |  |  |  | |  |  | |  |
| =< 200 | ref | ref | ref | ref | ref | ref | ref | | ref | ref | | ref |
| >200 to =<400 | 1.1[0.5-2.4] | 1.2[0.6-2.5] | 1.1[0.6-1.7] | 1.0[0.6-1.7] | 1.4[0.7-2.7] | 1.4[0.6-3.0] | 0.9[0.5-1.5] | | 1.0[0.6-1.6] | 1.4[0.9-2.2] | | 1.5[1.0-2.3] |
| >400 to =< 800 | 1.5[0.7-3.1] | 1.6[0.8-3.2] | 1.0[0.6-1.7] | 1.0[0.6-1.6] | 0.8[0.4-1.6] | 0.8[0.4-1.6] | 1.2[0.8-1.9] | | 1.3[0.8-1.9] | 1.5[1.0-2.3] | | **1.6*[1.1-2.3]** |

Note: RPR: relative prevalence ratio; ARPR: adjusted relative prevalence ratio; CI: confidence interval; m: meter; P: processed food; q: quintile; ref: reference category; UNP: unprocessed food.

^a^ Food including beverages.

^b^ Road-network distance in meters.

^c^ Multinomial regressions were conducted with the reference category being “Outlets selling mainly UNP foods”. In this table, unprocessed foods refer to unprocessed/minimally processed foods and processed culinary ingredients (NOVA groups 1 and 2). Processed foods refer to processed and ultra-processed foods (NOVA groups 3 and 4).

^d^ Models adjusted for all the variables presented in column one (i.e., type of school, poverty rates and total population of the areas where schools are located, governorates where schools are located, and distance from schools to food exposures).

^e^ Poverty rate (as percentage per capita) and population count (as total number of individuals) of each department of Greater Tunis were retrieved from a report produced by the National Office of Statistics of Tunisia, in collaboration with the World Bank ^(2)^. Poverty rates were categorized into tertiles as follows: High poverty rate (7.3-15.2%); Medium poverty rate (4.1-7.1%) and Low poverty rate (0.2-3.8%). Total population count was categorized into quintiles as follows: q1 (17408-27749 individuals); q2 (29185-40101); q3 (41830-57194); q4 (58792-84312) and q5 (86024-129693). Each school was matched to its corresponding department’s poverty rate tertile and population quintile.

^f^ Distance (road-network) in meters from schools to food retailers within buffer zones.

Numbers in **bold** indicate statistical significance: * p<.05; **p<.01; *** p<.001.

# **Supplementary table 4. Frequency and location of food advertisement sets around 50 primary schools in Greater Tunis**.

|  | **Food ^a^ advertisement sets within 800m ^b^ of schools** | | | | | |
| --- | --- | --- | --- | --- | --- | --- |
| **Location** | **Total** | | **Classification based on NOVA ^c^** | | | |
|  |  |  | **Healthy** | **Mixed** | **Unhealthy** | **Unclear ^d^** |
|  |  | **n ^e^** | | | | |
| **Billboards** | 21 | | 2 | 1 | 18 | 0 |
| **Storefront of** *^f^* |  | |  |  |  |  |
| *Butcher, poultry, and fish stores (n=243)* | 148 | | 62 | 70 | 9 | 7 |
| *Fruits and vegetables stores and markets (n=216)* | 71 | | 47 | 8 | 14 | 2 |
| *Mobile vendors selling mainly UNP foods^g^ (n=12)* | 0 | | 0 | 0 | 0 | 0 |
| *Hyper/Supermarkets (n=77)* | 10 | | 3 | 2 | 1 | 4 |
| *Mini markets (n=35)* | 22 | | 2 | 7 | 12 | 1 |
| *Corner shops/ “attar” (n=788)* | 578 | | 25 | 98 | 435 | 20 |
| *Full-service restaurants (n=47)* | 13 | | 4 | 4 | 4 | 1 |
| *Dairy stores (n=31)* | 24 | | 5 | 12 | 7 | 0 |
| *Local limited-service restaurants (n=586)* | 324 | | 9 | 47 | 247 | 21 |
| *International fast-food chains (n=3)* | 2 | | 0 | 0 | 2 | 0 |
| *Mobile vendors selling mainly P foods ^g^ (n=17)* | 5 | | 1 | 1 | 3 | 0 |
| *Coffee and tea restaurants (n=732)* | 267 | | 198 | 31 | 33 | 5 |
| *Desserts and cocktail places (n=23)* | 11 | | 0 | 2 | 7 | 2 |
| *Kiosks (n=590)* | 488 | | 9 | 75 | 382 | 22 |
| *Bakeries and pastries stores (n=221)* | 79 | | 0 | 10 | 68 | 1 |
| *Food wholesalers ^g^* | 35 | | 4 | 15 | 13 | 3 |
| **Total** | 2098 | | 371 | 383 | 1255 | 89 |

Note: m: meters.

^a^ Food including beverages.

^b^ Road-network distance in meters.

^c^ NOVA classification ^(1)^.

^d^ Unclear corresponds to food items that could not be categorized because (a) pictures were blurred or (b) it is not possible to deduce the NOVA-processing level of the food items included in the pictures ^(1)^.

^e^ Non-standardized counts were generated by summing the GIS data points within the 800m buffers across the 50 schools. For schools with overlapping buffers, GIS data points were included in the count of each school.

^f^ Numbers in parenthesis indicate total number of food retailers by type.

^g^ Mobile vendors include (a) vendors selling unprocessed/minimally processed foods such as vegetables, fruits, and popcorn and (b) vendors selling processed/ultra-processed foods such as sandwiches, carbonated beverages, and crepes.

^g^ Wholesalers were excluded from retailer counts but their storefront advertisements were included in advertisement counts.

# **Supplementary table 5. Associations between type of food advertisement-sets and school characteristics across primary schools in Greater Tunis.**

| **Food ^a^ advertisement sets within 800 m^b^ of schools** | **Units of analysis:**  **GIS data points ^c^** | | | | **Multinomial regression** ^d^ (Reference category: Healthy) | | | |
| --- | --- | --- | --- | --- | --- | --- | --- | --- |
|  | **Healthy**  **(n=371)** | **Mixed**  **(n=383)** | | **Unhealthy**  **(n=1255)** | **Mixed** | | **Unhealthy** | |
|  | **n** | | | | **RPR [95% CI]** | **ARPR^e^ [95% CI]** | **RPR [95% CI]** | **ARPR^e^ [95% CI]** |
| **Type of school** | | | | | | | | |
| Public | 270 | | 256 | 941 | ref | ref | ref | ref |
| Private | 101 | | 127 | 314 | 1.3[0.9-1.8] | 1.1[0.8-1.6] | 0.9[0.7-1.2] | 0.8[0.7-1.1] |
| **Poverty rate of the departments where school are located ^f^ (tertiles)** |  | | | |  | |  | |
| High poverty rate | 69 | | 62 | 233 | ref | ref | ref | ref |
| Medium poverty rate | 155 | | 162 | 50 | 1.1[0.6-2.0] | 0.7[0.4-1.2] | 1.0[0.7-1.5] | 0.9[0.6-1.4] |
| Low poverty rate | 147 | | 159 | 472 | 1.1[0.7-2.0] | 0.8[0.5-1.2] | 0.9[0.6-1.3] | 0.8[0.6-1.2] |
| **Total population count of the departments where schools are located ^f^ (quintiles)** |  | | | |  | |  | |
| q1 | 42 | | 50 | 161 | ref | ref | ref | ref |
| q2 | 80 | | 74 | 227 | 0.7[0.4-1.2] | 0.7[0.4-1.1] | 0.7[0.5-1.0] | 0.7[0.5-1.0] |
| q3 | 46 | | 51 | 175 | 0.9[0.5-1.7] | 0.8[0.4-1.6] | 1.0[0.7-1.5] | 1.0[0.7-1.6] |
| q4 | 105 | | 84 | 310 | 0.7[0.4-1.3] | 0.6[0.3-1.3] | 0.8[0.5-1.1] | 0.8[0.5-1.2] |
| q5 | 98 | | 124 | 382 | 1.0[0.6-1.8] | 1.2[0.7-1.9] | 1.0[0.7-1.5] | 1.2[0.8-1.9] |
| **Governorates where schools are located** |  | | | |  | |  | |
| Tunis | 180 | | 207 | 640 | ref | ref | ref | ref |
| Ariana | 64 | | 48 | 199 | **0.7[0.5-0.9]*** | **0.5[0.3-0.7]***** | 0.9[0.6-1.2] | 0.7[0.5-1.0] |
| Ben Arous | 94 | | 107 | 314 | 1.0[0.6-1.5] | 1.0[0.7-1.5] | 0.9[0.7-1.3] | 1.0[0.7-1.3] |
| Manouba | 33 | | 21 | 102 | 0.6[0.2-1.4] | 0.6[0.2-1.3] | 0.9[0.6-1.3] | 0.9[0.5-1.4] |
| **Distance from school to food advertisement sets (m)^g^** |  | |  |  |  |  |  |  |
| =< 200 | 40 | | 47 | 123 | ref | ref | ref | ref |
| >200 to =<400 | 93 | | 80 | 292 | 0.7[0.4-1.3] | 0.7[0.4-1.2] | 1.0[0.6-1.7] | 1.0[0.6-1.6] |
| >400 to =< 800 | 238 | | 256 | 840 | 0.9[0.6-1.5] | 0.9[0.5-1.5] | 1.2[0.8-1.9] | 1.2[0.8-1.9] |

Note: RPR: relative prevalence ratio; ARPR: adjusted relative prevalence ratio; CI: confidence interval; GIS: Geographic Information System; m: meters; q: quintile; ref: reference category.

^a^ Food including beverages.

^b^ Road-network distance in meters.

^c^ Non-standardized counts were generated by summing the GIS data points within the 800m buffers across the 50 schools. For schools with overlapping buffers, GIS data points were included in the count of each school.

^d^ Multinomial regressions were conducted with the reference category being ‘Healthy’ food outlets.

^e^ Models adjusted for all the variables presented in column one (i.e., type of school, poverty rates and total population of the areas where schools are located, governorates where schools are located, and distance from schools to food exposures).

^f^ Poverty rate (as percentage per capita) and population count (as total number of individuals) of each department of Greater Tunis were retrieved from a report produced by the National Office of Statistics of Tunisia, in collaboration with the World Bank ^(2)^. Poverty rates were categorized into tertiles as follows: High poverty rate (7.3-15.2%); Medium poverty rate (4.1-7.1%) and Low poverty rate (0.2-3.8%). Total population count was categorized into quintiles as follows: q1 (17408-27749 individuals); q2 (29185-40101); q3 (41830-57194); q4 (58792-84312) and q5 (86024-129693). Each school was matched to its corresponding department’s poverty rate tertile and population quintile.

^g^ Distance (road-network) in meters from school to food advertisement sets within each buffer.

Numbers in **bold** indicate statistical significance: * p<.05; **p<.01; *** p<.001.


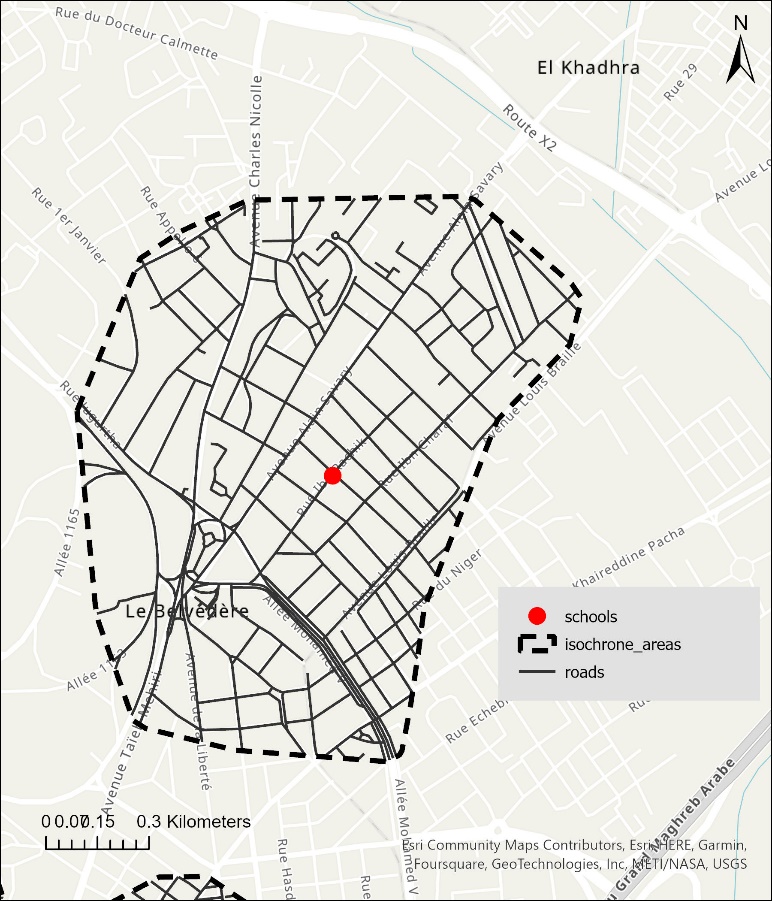


# **Supplementary figure 2. Sample map of an 800-meter road network buffer around one school (service area polygon).**

*Legend: This road-network buffer was obtained by drawing all the roads corresponding to an 800m walking distance from school entrance, and then connecting the endpoints of all these roads to create a polygon.*


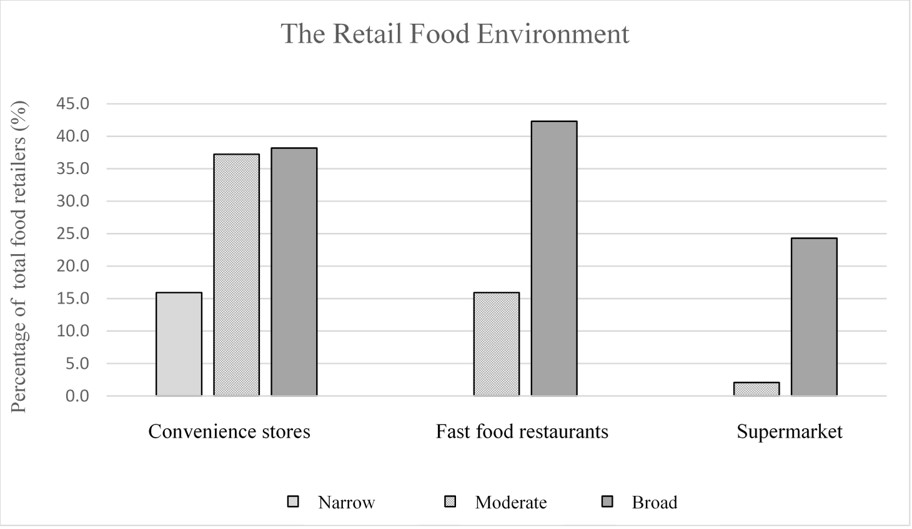


# **Supplementary figure 3**. **The retail food environments in Greater Tunis using different construct definitions.**

*Legend: Density of convenience stores, fast food restaurants, and supermarkets (as a percentage of total food retailers) using the various construct definitions developed by Wilkins et al, 2019 ^(3)^.*

*According to Wilkins et al, 2019 ^(3)^: For convenience stores, the “narrow” definition includes small stores selling minimal or no fresh goods; “moderate” includes narrow convenience stores plus stores selling a wider but still limited range of fresh good; and “broad” includes moderate convenience stores plus small grocery stores. For fast food restaurants, the “moderate” definition includes chain and non-chain fast-food restaurants and “broad” includes moderate fast food restaurants plus retail bakeries, takeaway cafes, and chain coffee shops. For supermarkets, the “moderate” definition includes small and large supermarkets; and “brood” includes supermarkets and small grocery stores.*

# **References**

1. Monteiro CA, Cannon G, Levy R *et al.* (2016) NOVA. The star shines bright. *World Nutrition* 7, 28-38.

2. National Institute of Statistics in Tunisia and World-Bank (2020) Carte de La Pauvreté En Tunisie. <http://ins.tn/sites/default/files/publication/pdf/Carte%20de%20la%20pauvret%C3%A9%20en%20Tunisie_final_0.pdf> (accessed 10 October 2022)

3. Wilkins E, Morris M, Radley D *et al.* (2019) Methods of measuring associations between the retail food environment and weight status: importance of classifications and metrics. *SSM-population health* 8, 100404.
